# Prevalence and correlates of apathy in myotonic dystrophy type 1

**DOI:** 10.1186/s12883-015-0401-6

**Published:** 2015-08-22

**Authors:** Benjamin Gallais, Michèle Montreuil, Marcela Gargiulo, Bruno Eymard, Cynthia Gagnon, Luc Laberge

**Affiliations:** Department of Neuromuscular Disorders, CHU Salpêtrière, Paris, France; Laboratoire Psychopathologie et Neuropsychologie, Université Paris 8, Paris, France; Groupe de recherche interdisciplinaire sur les maladies neuromusculaires (GRIMN), CSSS de Jonquière, 2230 rue de l’Hôpital, G7X 7X2 Saguenay, Québec Canada; Faculté de médecine et des sciences de la santé, Université de Sherbrooke, Sherbrooke, Québec, Canada; ÉCOBES – Recherche et transfert, Cégep de Jonquière, Saguenay, Québec Canada; Département des sciences de la santé, Université du Québec à Chicoutimi, Saguenay, Québec Canada

## Abstract

**Background:**

Apathy in DM1 has long been acknowledged in clinical practice. However, a major drawback is that the concept has been only sparsely explored in previous specific studies. This study aimed to determine the prevalence of apathy in myotonic dystrophy (DM1), to compare it with facioscapulohumeral dystrophy (FSHD) patients and normal healthy controls, and explore its relationship to psychopathological features and cognitive function.

**Methods:**

Levels of apathy in 38 DM1 patients with adult phenotypes were compared with 19 patients with FSHD and 20 matched controls. Patient participants were consecutively recruited, regarding their interdisciplinary annual evaluation at the neuromuscular pathology reference center (Institute of Myology, Paris, France), within an 18-month period. Additional measurements included motor disability, fatigue, depression, anxiety, and cognitive abilities. Inter-group comparisons were performed using non-parametric Kruskal-Wallis tests and Mann–Whitney U Tests. Intra-group comparisons were carried out with the Wilcoxon Signed rank and Friedman tests. Also, Spearman’s correlations were used to assess the strength of linear relationships between pairs of variables. The significance level was set at 0.05.

**Results:**

Global score of apathy was significantly higher in DM1 patients than in FSHD patients (*p* < 0.01) and in controls (*p* < 0.001). Sixteen of 38 DM1 patients (39.5 %) met the criterion for apathy, contrasting with only 4 of the 19 (21.1 %) FSHD patients. No control subject was apathetic. Moreover, apathy in DM1 patients was negatively correlated to MMSE (*r* = −.46, *p* < .05) and Stroop Word (*r* = −.55, *p* < .01) scores, but not with age, educational level, disease duration, CTG repeats, motor functional disability, fatigue, depression, and anxiety.

**Conclusions:**

Apathy is a frequent symptom in DM1 (almost 40 %). It is more prevalent than in a similarly disabled group of patients with FSHD and in controls. Results also show that apathy in DM1 is independent of the psychopathological domain, fatigue, age, and motor disability, but associated to general cognitive status. These results altogether could suggest a central cause for apathy in DM1 rather than an adjustment process to cope with the progressive and debilitating nature of the disease. Data emphasize the importance to evaluate this symptom in routine clinical management of DM1 patients.

**Electronic supplementary material:**

The online version of this article (doi:10.1186/s12883-015-0401-6) contains supplementary material, which is available to authorized users.

## Background

Myotonic dystrophy type 1 (DM1) is a multisystemic, hereditary disease affecting the muscular, central nervous, ocular, respiratory, cardiovascular, digestive, endocrine and reproductive systems [1, 2]. It is caused by an unstable base triplet (CTG)n repeat located in the 3′ untranslated region of the dystrophia myotonica protein kinase (*DMPK*) gene on chromosome 19 [3]. DM1 is divided into four different phenotypes according to age at onset of symptoms: congenital; childhood; classical or adult form; and late onset form [4].

The degree of cognitive impairment varies with the phenotype, ranging from mental retardation and learning difficulties in the congenital [1] and childhood phenotypes [5] to neuropsychological impairments affecting the higher cognitive functions in the classical adult phenotype. More particularly, concentration and attention problems, deficits in visuo-spatial and visuo-constructive skills, and a tendency to rigidity and perseveration [6, 7] were noted in patients with this latter phenotype.

Studies have revealed that personality traits of patients with adult onset phenotypes were characterized by pervasive social inhibition and avoidance of social interaction [8]. For example, low scores on the self-directedness and cooperativeness subscales of the Temperament and Character Inventory (TCI) were reported, respectively reflecting feelings of ineptitude when faced with obstacles and difficulties in accomplishing goals and a tendency to prefer solitude to company and poor functioning in social groups [9].

Also, Rohrer was the first to note apathy and a lack of motivation in patients with DM1 [10], although Curschmann (cited in Thomasen [11]) had previously evoked dullness as a characteristic component of the condition. Thomasen later identified fatigue and adynamia as prominent symptoms of DM1 patients and pointed out the difficulty to distinguish between true fatigue and lack of initiative characteristic of mental change [11]. Bungener et al. further underlined a significant emotional deficit in this patient population, manifested by a lack of expressiveness, a monotonous mood, an inability to anticipate pleasure, and apathy, without major depressive episode [12]. In their landmark study on 36 DM1 and 13 Charcot-Marie-Tooth (CMT) patients, Rubinsztein and colleagues [13] specified that apathy was more severe in patients affected by DM1 than in those affected by CMT disease. Unfortunately, the authors did not report the prevalence of apathy per se. In addition, they found that apathy did not vary with duration of illness, muscular weakness, depression, daytime sleepiness, and fatigue. The absence of relationship between apathy and depression was also noted in a subsequent smaller study (*n* = 10 DM1 patients) looking at an anthiarrhytmic drug [14]. No study has though explored the relationship of apathy to cognitive abilities in DM1 patients.

This study aims to determine the prevalence of apathy in DM1 patients and to compare it with that found in patients affected by facio-scapulo-humeral dystrophy (FSHD) and controls. FSHD has been chosen as a comparative group because it shares many features with the classic adult phenotype of DM1, including autosomal dominant inheritance, age of onset, rate of disease progression, and broad correlation between the mutation repetitions and disease severity. On the other hand, FSHD patients do not generally exhibit cognitive dysfunction, except in patients with very small size of the deleted fragment [15]. A secondary objective of this study is to explore the relationship of apathy to psychopathological features, cognitive functions, fatigue, and functional motor disability in DM1.

## Methods

### Participants

Patients with both DM1 and FSHD were recruited consecutively from the National Reference Center for diagnosis and follow-up of neuromuscular disorders at the Pitié-Salpêtrière Hospital (Paris, France). During an 18-month period, 39 DM1 and 22 FSHD inpatients were invited to participate in the study. One DM1 and 3 FSHD patients refused to participate. Inclusion criteria were: 1) age 18–70, 2) no history of major psychiatric or other somatic illness, acquired brain injury or alcohol or drug abuse, and 3) a molecular confirmation of the DM1 or FSHD diagnosis. Patients with congenital or childhood DM1 phenotypes were excluded [16]. A total of 38 patients with adult or late-onset phenotypes of DM1 (22 women and 16 men; mean age (SD), 36.8 (10.2); age range, 21–60 years) and 19 FSHD (10 women and 9 men; mean age (SD), 46.0 (13.5); age range, 20–66 years) completed the study. Controls included 20 healthy volunteers (16 women and 4 men; mean age (SD), 38.6 (17.8); age range, 18–70). DM1 patients and control subjects were matched by age and educational level. Since socioeconomic and educational levels can be considered as important features in DM1, we precisely defined the working classes of the DM1 patients, and then, matched 2 or 1 DM1 subjects with 1 control with the same working class and approximatively the same age.

Written informed consent was obtained from all participants after the research project was fully explained. In accordance with the policy in the context of an academic research with minimal risks for participants at the time of the study, the Ethics Committee of the University of Paris-Vincennes approved the study design and procedures.

### Instruments

The 1-h research protocol was administered by a trained psychologist.

### Demographic data

The questionnaire included age, gender, and number of completed years of schooling.

### Functional disability assessment

Functional disability was assessed using the Walton Functional Scale [17]. Scores range from 0 to 10, with a higher score representing a higher degree of disability.

### Apathy and psychopathological assessment

Apathy was assessed with the Lille Apathy Rating Scale (LARS). The LARS is a 33-item semi-structured and standardized interview tapping nine domains (i.e., everyday productivity, interests, taking initiative, novelty seeking, voluntary actions, emotional responses, concern, social life, and self-awareness). Global LARS scores range from −36 to +36, with higher scores corresponding to greater apathy. The recommended cut-off score for apathy is −16. The LARS has shown satisfactory inter-rater and test–retest reliability as well as excellent concurrent and criterion related validity [18]. Its four factorial sub-scores permits to assess the following dimensions: intellectual curiosity (LARS-IC), emotion (LARS-E), action initiation (LARS-AI), and self-awareness (LARS-SA). Theoretically, LARS-IC, LARS-E and LARS-AI relate to the cognitive, emotional, and behavioural goal-directed dimensions of apathy.

The Mini International Neuropsychiatric Interview was used to diagnose current major depressive episodes (MDE) [19]. Severity of depressive symptoms was assessed using the Montgomery and Asberg Depression Rating Scale (MADRS) [20]. Scores ≥ 15 are indicative of depression.

The State-Trait Anxiety Inventory (STAI) for Adults was used to evaluate both temporary condition of “state anxiety” and the more general and long-standing quality of “trait anxiety” [21]. Raw scores are transformed in “T-Scores” corresponding to the equivalent normative score, depending on type of anxiety (state or trait) and gender of subject (female and male). T-scores over 55 and 65 are considered as high and very high levels of anxiety.

### Cognitive assessment

All subjects underwent a formal neuropsychological testing battery. The Mini Mental State Evaluation (MMSE) was used to estimate the severity of cognitive impairment [22]. The Stroop Test (Color and Word Stroop test, [23]) was used to evaluate processing speed, attentional control, and response inhibition [24]. It consists of 3 subtests, namely word reading, color naming, and color-word interference. The Trail Making Test A and B (TMT) was used to evaluate cognitive flexibility [25]. Finally, the Frontal Assessment Battery (FAB) was used to evaluate executive abilities, namely conceptualization, mental flexibility, programming, sensitivity to interference, inhibitory control, and environmental autonomy [26].

### Fatigue assessment

Fatigue was assessed with the Krupp’s Fatigue Severity Scale (KFSS) which consists of 9 questions on a 7-point Likert scale [27]. Scores of ≥4 are indicative of fatigue.

### Statistical analyses

Inter-group comparisons were performed using non-parametric Kruskal-Wallis tests to compare the scores in the different tests and questionnaires between the three groups, and Mann–Whitney U Tests for post-hoc analyses. Additionally, a Pearson’s chi-square test was performed to determine whether there was a significant difference between the frequencies of apathetic subjects in the different groups. Intra-group comparisons were carried out with the Wilcoxon Signed rank and Friedman tests. Also, Spearman’s correlations were used to assess the relationship between apathy and its possible related factors. The significance level was set at 0.05. SPSS 18.0 was used for statistical analyses.

## Results

Socio-demographic data of DM1 and FSHD patients and controls are shown in Table 1. FSHD patients were older than DM1 patients (*p* = 0.004).

**Table 1:** Demographic and clinical characteristics of DM1, FSHD and control subjects

Characteristics	DM1	FSHD	Controls
N	38	19	20
Socio-demographic			
Age in years, mean (SD)	36.0 (10.2)^a^	46.0 (13.5)	38.6 (17.8)
Range in years	21–60	20–66	18–70
Educational level in years, mean (SD)	10.8 (3.6)	11.7 (4.7)	10.9 (3.1)
Clinical			
CTG repeats, mean (SD)	568 (341)	─	─
*KpnI* repeats, mean (SD)	─	6.17	─
Disease duration, mean (SD)	16.0 (8.8)	22.4 (12.5)	─
Walton Functional Scale (SD)	2.13 (1.6)	3.16 (2.1)	─

^a^Significant difference between DM1 and FSHD, *p* < 0.05

### Apathy

The global score of apathy was higher in DM1 patients than in FSHD patients (*p* < 0.006) and controls (*p* = 0.000) (Table 2). Also, FSHD patients did not differ from controls regarding apathy global score. In addition, 16 out of 38 DM1 patients (39.5 %) met the criterion for apathy. In comparison, only 4 out of 19 FSHD (21.1 %) patients met the criterion for apathy. A Pearson’s chi-square test indicates that this difference observed in the proportions of apathetic subjects did not reach the level of significance (*p* = .164). No control subject was apathetic.

**Table 2:** Psychopathological and neuropsychological results of DM1, FSHD and control subjects

Characteristics	DM1	FSHD	Controls
N	38	19	20
Psychopathology			
Apathy, %	39.5	21.1	0
LARS, mean (SD)	−16.9 (8,5)^ba^	−22.8 (6.3)	−25.4 (4.8)
LARS-IC, mean (SD)	−1.53 (1.2)^ba^	−2.45 (.7)	−2.7 (.7)
LARS-E, mean (SD)	−2.1 (1.4)^a^	−2.66 (1.2)	−2.9 (1.2)
LARS-AI, mean (SD)	−2.44 (1.1)^a^	−2.73 (1.1)	−3.05 (.7)
LARS-SA, mean (SD)	−1.79 (2.1)^a^	−2.3 (1.6)	−2.21 (1.7)
MADRS, mean (SD)	10.3 (8.8)	7.0 (7.4)	6.1 (6.2)
STAI-A, T score, mean (SD)	50.1 (14.1)	44.8 (11)	45.3 (9)
STAI-B, T score, mean (SD)	53.3 (12.6)	48.7 (12.9)	47.1 (10.9)
KFSS, mean (SD)	4.6 (1.5)^a^	4.8 (1.5)^c^	3.3 (1.0)
Neuropsychology			
MMSE, mean (SD)	27.5 (2.2)^ba^	28.9 (0.9)	29.3 (0.9)
FAB, mean (SD)	15.5 (1.7)^a^	15.9 (1.4)	16.5 (0.95)
Stroop word T score, mean (SD)	47.8 (6.6)	51.6 (8.9)	51.8 (7)
Stroop color T score, mean (SD)	44.24 (8.2)	46.56 (9.8)	49.75 (6.3)
Stroop color-word T score, mean (SD)	39.0 (9.9)^ba^	49.2 (7.9)	51.8 (4.9)
Stroop interference score, mean (SD)	44.47 (8.4)^ba^	49.68 (6.5)	50.65 (6.6)
TMT-A z score, mean (SD)	0.2 (0.7)^ba^	0.9 (0.4)	1.0 (0.5)
TMT-B z score, mean (SD)	−0.4 (1.2)^ba^	0.4 (0.6)	0.4 (0.7)

*LARS-IC* LARS Intellectual curiosity, *LARS-E* LARS Emotion, *LARS-AI* LARS Action initiation, *LARS-SA* LARS Self-awareness, *MADRS* Montgomery Asberg Depression Rating Scale, *STAI-A* state anxiety, *STAI-B* trait anxiety, *KFSS* Krupp fatigue severity scale, *MMSE* Mini Mental State Evaluation, *FAB* Frontal Assessment Battery, *TMT* Trail Making Test

^a^Significant difference between DM1 and Controls, *p* < 0.05

^b^Significant difference between DM1 and FSHD, *p* < 0.05

^c^Significant difference between FSHD and Controls, *p* < 0.05

A difference between DM1 and FSHD patients was noted in the “Intellectual Curiosity” (*p* = 0.002) domain. In addition, differences were noted between DM1 and controls in the “Intellectual Curiosity” (*p* = 0.0000), “Emotion” (*p* = 0.020), “Action initiation” (*p* = 0.030), and “Self-awareness” (*p* = 0.044) domains. No difference was found between FSHD and control subjects relatively to LARS sub-scores (Table 2).

### Psychopathology

Nine DM1 (23.7 %) and 1 FSHD (5.3 %) patients met the criteria of current MDE (MINI). No control subject was clinically depressed. No between group differences was observed as regards severity of depressive symptoms (MADRS).

Mean state anxiety scores of DM1 subjects (Table 2) are indicative of moderate anxiety while those of FSHD patients and controls are indicative of mild anxiety. Mean trait anxiety scores of all groups are indicative of moderate anxiety. Moreover, no between group differences was observed in severity of state and trait anxiety (Table 2).

### Neuropsychological features

A difference between DM1 and FSHD patients was observed for MMSE (*p* = 0.028), Stroop Color-Word (*p* = 0.001), Stroop Interference (*p* = 0.011), TMT-A (*p* = 0.000), and TMT-B scores (*p* = 0.004). Also, DM1 and controls differed as regards MMSE (*p* = 0.001), FAB (*p* = 0.015), Stroop Color-Word (*p* = 0.000), Stroop Interference (*p* = 0.003), TMT-A (*p* = 0.000), and TMT-B scores (*p* = 0.003) (Table 2).

### Fatigue

DM1 and FSHD patients reported higher levels of fatigue than control (respectively *p* = 0.004 and *p* = 0.003) (Table 2).

### Relationships between apathy and clinical, psychopathological, and cognitive features in DM1

No correlation was found between apathy and age, educational level, disease duration, CTG repeats, and motor functional disability (Table 3). A Mann–Whitney *U* test revealed no difference in the apathy levels of male and female DM1 patients.

**Table 3:** Correlation between apathy and sociodemographic, clinical, psychopathological and neuropsychological variables in DM1 patients

	LARS-Total Score	LARS-IC	LARS-E	LARS-AI	LARS-SA
Age	.130	.116	.188	−.183	.152
CTG	−.024	.137	−.188	−.198	.114
DiseaseDuration	.049	.076	.069	−.335^*^	.092
Educational level	−.135	−.288	.157	−.197	.023
Walton	.166	.088	.095	−.127	.234
MADRS	.005	.161	−.094	−.093	−.327^*^
StaiA	.013	.214	−.045	−.221	−.451^**^
StaiB	.056	.265	−.145	−.102	−.367^*^
MMSE	−.464^*^	−.457^**^	−.042	−.032	−.130
FAB-TotalScore	−.006	−.090	.135	−.031	.080
FAB-Similarities	.128	.258	−.013	−.123	−.042
FAB-Lex.Fluency	−.088	−.297	.018	−.001	.316
FAB-Prehension	−.278	−.305	.003	−.364^*^	.027
FAB-MotorSeries	−.097	−.167	−.077	.037	.020
FAB-Conflict	.028	−.045	.162	.023	−.119
FAB-GoNogo	−.125	−.167	−.156	.111	−.239
StroopWord	−.548^**^	−.253	−.372^*^	−.221	−.092
StroopColor	−.091	−.060	−.093	−.204	−.060
StroopColorWord	−.097	−.057	−.138	.038	−.145
StroopInterfer	.060	−.016	−.009	.212	.021
TMTA	−.166	−.044	−.067	.003	−.014
TMTB	−.226	−.172	−.279	.039	−.252
KFSS	.068	.095	−.187	.025	−.108

Spearman’s correlation

*LARS.IC* LARS Intellectual curiosity, *LARS-E* LARS Emotion, *LARS-AI* LARS Action initiation, *LARS-SA* LARS Self-awareness, *MADRS* Montgomery Asberg Depression Rating Scale, *MMSE* Mini Mental State Evaluation; *FAB* Frontal Assessment Battery, *TMT* Trail Making Test, *KFSS* Krupp fatigue severity scale

**p < 0.01

*p < 0.05

Moreover, apathy was not correlated with fatigue, depression and state or trait anxiety. Additionally, no corelation was found between apathy and level of depression in the 9 subjects with a current MDE (see Additional file 1: Table S1). Contrastively, apathy levels were negatively correlated to MMSE (*r* = −.46, *p* = 0.019) and Stroop Word (*r* = −.55, *p* = 0.005) scores.

## Discussion

In the general population, apathy is often defined as an absence or lack of feeling, emotion, interest or concern. Marin [28] has stressed that the lack of motivation inherent to apathy is clinically expressed as reduced goal-directed behavior, cognition, and emotion. He further proposed that such features are not attributable to diminished level of consciousness, cognitive impairment or emotional distress.

Apathy in DM1 has long been acknowledged in clinical practice. However, a major drawback is that the concept has been only sparsely explored in previous specific studies [29]. This study demonstrates that apathy is highly prevalent in DM1 patients compared to matched healthy controls. Apathy is also more frequent in DM1 than in a similarly disabled group of patients with FSHD, despite a statistical non-significance in proportions, suggesting that this symptom is not only consecutive to some adjustment process to cope with a chronic progressive neuromuscular disorder [13].

Findings also confirm the absence of relationship between apathy and age, gender, disease duration, CTG repeats, functional impairment, and fatigue in DM1 patients [13, 14]. The absence of a significant correlation between apathy and fatigue must be pointed out considering the conceptual proximity of lack of motivation and experienced fatigue [30].

### Which aetiology for apathy in DM1?

#### The emotional hypothesis

Apathy can easily be confounded with depression, overlapping with diminished interest, and loss of energy, but it would be simplistic to reduce apathy to depression. In the present study, no correlation was found between apathy and depression, as reported elsewhere [13, 14]. Studies in other neurological diseases have also concluded that apathy is a separate entity from depression [18, 31, 32]. Levy and Dubois have stated that: “… apathy is a symptom that can be observed in depression but may also occur without depression and, when both are present in a given patient, they may be clinically and anatomically independent” [33].

In the present study, 10 DM1 patients were apathetic but not depressed while 4 were only depressed.

Levy et al. [31] further suggested that the diagnosis of depression should be supported by symptoms of sadness and feelings of helplessness, hopelessness, and worthlessness that should not be present in patients who are only apathetic.

In view of the present results, one may advise clinicians to systematically evaluate both these symptoms since treatment options may differ. Before classical cognitive and behavioural therapy of depression is undertaken, clinicians should also know whether a patient is apathetic or not as self-participation into the protocol is highly recommended to reach successful outcomes.

There are still unresolved differences of opinions as to whether depression in DM1 is a direct result of brain dysfunction or a secondary reaction to the physical disability or a combination of both [34]. Some authors have advanced that the relatively high prevalence of depression of mild to moderate intensity observed in DM1 [34] is partly attributable to symptoms of apathy, such as lack of motivation, blunted emotional concerns and reactions [12, 14]. Our results are in agreement with this position. Indeed, even though 1 of 4 DM1 patients is clinically depressed, their level of depression does not significantly differ from FSHD patients and controls. Thus, there is a distinct possibility that the profile of monotonous affect and lack of emotional expressiveness repeatedly described in DM1 is more likely explained by apathy than pure depression. Moreover, the lack of facial expression due to weakness of facial muscles characteristic of DM1 may mimic depressive symptoms such as emotional expressiveness, sadness or anhedonia, leading to possible biases in diagnosis of major depressive episodes in DM1 [34]. Finally, an overlap between the “somatic dimension” of depression and classical symptoms of DM1 such as fatigue, sleep disturbances, or even reduced appetite has been described and may explain high frequency of depression diagnoses [34].

#### The “central” hypothesis

Results revealed that apathy was associated with the general cognitive status and word reading abilities, supporting the view that apathy reflects a central nervous system involvement [13] in DM1 patients. This is consistent with previous findings in patients with other neurological conditions in whom lower global intellectual status was associated with higher levels of apathy [31, 32, 35]. Further studies may evaluate the correlation of apathy with more extended intelligence tests battery in DM1 (e.g., the Wechsler Adult Intelligence Scale). Moreover, apathy is related to a dysfunction of frontal-subcortical circuits, following direct lesion of the prefrontal cortex or after lesions of basal ganglia structures [33]. More particularly, Varanese et al. found a positive relationship between apathy and executive dysfunction levels [36].

Results yet revealed weak relationships between levels of apathy and scores on executive function tests (coefficients varying between −0.006 and − .278). Previous studies in other neurological diseases have shown that apathy was associated with poor planning and rule-finding [36] as well as high numbers of rule breaks [37], with tasks requiring great levels of executive functioning. It should though be underlined that these latter aspects of executive functioning cannot be assessed with fast and easy-to-use tests such as the ones used in the present study. Other studies must verify whether brain microstructural changes in fronto-subcortical circuits are associated with apathy in DM1 patients, as it was demonstrated in other neurological diseases [38].

### Clinical relevance of apathy dimensions

This study further specifies that dimensions of apathy differ between DM1 patients and controls, in agreement with previous observations in DM1. The emotional deficit manifested by a lack of expressiveness and monotonous mood [12] may be due to emotional apathy (LARS-E), characterised by a blunting of emotional responses and lack of concern [18]; lower social participation, namely difficulties in carrying out daily activities and social roles [39] may be partly attributed to the apathy’s behavioural (LARS-AI) [35] and cognitive dimensions (LARS-IC & LARS-SA), reflecting respectively lack of initiative and low everyday productivity and low interest in novelty as well as drop in the perceived need for knowledge [18]; finally, high scores in all four dimensions of apathy could explain, at least partly, the lack of compliance/observance in care frequently observed in DM1 patients [40].

### Study limitations

Since the recruitment of subject was consecutive, the selection bias should be small, as it was based on the interdisciplinary annual evaluation and not on the basis of a dependent variable-specificity (*i.e.,* patients reporting apathy or cognitive complaints). Additionally, because of the relatively small sample size and the numerous variables entered into the statistical model, results have to be interpreted with caution. Limitations include the fact that the LARS is a patient-based tool for which individuals are required to use their own standards to evaluate their apathy and is thus subjective to response bias. Nevertheless, since LARS is a structured interview, clinicians can adjust their rating regarding their own perception of the verbal and non-verbal patients’ inputs. Future studies may add the LARS caregiver-based version [41], which was not yet published when this study was performed. Furthermore, FSHD patients were older than DM1. Because of the rareness of the disease, this factor has not been controlled. Since apathy may occur in dementia, a link between age and apathy may be supposed. Yet, age was not correlated to apathy in our results. Thus, the fact that DM1 patients were more apathetic than FSHD patients enhances the lack of relation between age and apathy.

Also, the neuropsychological battery used here does not consider the demands of the *real world* in regards to daily life barriers encountered by DM1 patients, such as “multitasking” situations for which apathy is likely to appear preferentially [37]. Further studies combining neuropsychological and psychopathological features of DM1 patients and their implications into psycho-social interactions are thus needed. Moreover, studies should use measurement instruments that are sensitive to the level of accomplishment of instrumental activities of daily living [42], in terms of both executive processes and motivational aspects involved in goal-directed behavioural, cognitive and emotional activities (e.g., the Gambling task [43] or the multiple errands shopping test [44]). Finally, further studies on apathy in DM1 should also aim at describing its progression and test experimental treatments.

## Conclusions

This is the first study of prevalence to show that apathy is more frequent in DM1 than in healthy normal subjects and in another neuromuscular disorder, namely the FSHD. Among the apathetic DM1 patients, the severity of their symptoms may constitute an additional barrier to patients’ social involvement. Since apathy has been described as the main source of complaint and psychological distress in neurologic patients’ family or caregivers [45], a better comprehension of apathy and its outcomes in DM1 is relevant. In this regard, a clinical routine evaluation of apathy in DM1 is advised.

In addition, results imply that apathy is independent of the psychopathological domain, fatigue, sleepiness, age, gender and motor disability, contrasting with its negative relationships with some of the neuropsychological indices. These results altogether could suggest a central cause for apathy in DM1 rather than an adjustment process to cope with the progressive and debilitating nature of the disease.

## Additional file

Additional file 1: Table S1. Correlation between apathy and depression in DM1 patients with a current major depressive episode (*n* = 9). (DOCX 15 kb)

## Abbreviations

DM1: Myotonic dystrophy type 1
CTG: Cytosine-thymine-guanine
CMT: Charcot-Marie-Tooth
FSHD: Facio-scapulo-humeral dystrophy
LARS: Lille Apathy Rating Scale
LARS-IC: LARS- intellectual curiosity dimension
LARS-E: LARS-emotion dimension
LARS-AI: LARS- action initiation dimension
LARS-SA: LARS-self-awareness dimension
MDE: Major depressive episode
MADRS: Montgomery and Asberg Depression Rating Scale
STAI: State-Trait Anxiety Inventory
MMSE: Mini Mental State Evaluation
TMT: Trail Making Test
FAB: Frontal Assessment Battery
KFSS: Krupp’s Fatigue Severity Scale
SPSS: Statistical Package for the Social Sciences
