# Supplementary material for: Prevalence and correlates of apathy in myotonic dystrophy type 1
Source: BMC Neurol. 2015 Aug 22;15:148. doi: 10.1186/s12883-015-0401-6 (PMC4546188; doi:10.1186/s12883-015-0401-6)
Supplement: Additional file 1: Table S1. — Correlation between apathy and depression in DM1 patients with a current major depressive episode (n = 9). (DOCX 15 kb) [file 12883_2015_401_MOESM1_ESM.docx]

**Table S1: Correlation between apathy and depression in DM1 patients with a current major depressive episode (n= 9)**

|  | **LARS-Total Score** | **LARS-IC** | **LARS-E** | **LARS-AI** | **LARS-SA** |
| --- | --- | --- | --- | --- | --- |
| MADRS | -.556 | -.571 | -.574 | -.483 | -.266 |

Spearman’s correlation. LARS-IC = LARS Intellectual curiosity; LARS-E = LARS Emotion; LARS-AI = LARS Action initiation; LARS-SA = LARS Self-awareness; MADRS = Montgomery Asberg Depression Rating Scale
